# Prediction of 2-[^18^F]FDG PET-CT SUVmax for Adrenal Mass Characterization: A CT Radiomics Feasibility Study

**DOI:** 10.3390/cancers15133439

**Published:** 2023-06-30

**Authors:** Arnaldo Stanzione, Renato Cuocolo, Claudia Bombace, Ilaria Pesce, Ciro Gabriele Mainolfi, Marco De Giorgi, Gregorio Delli Paoli, Pasquale La Selva, Jessica Petrone, Luigi Camera, Michele Klain, Silvana Del Vecchio, Alberto Cuocolo, Simone Maurea

**Affiliations:** 1 Department of Advanced Biomedical Sciences, University of Naples “Federico II”, 80131 Naples, Italy; 2 Department of Medicine, Surgery and Dentistry, University of Salerno, 84084 Baronissi, Italy

**Keywords:** PET-CT, radiomics, adrenal glands, SUVmax, neoplasms

## Abstract

**Simple Summary:**

Adrenal masses represent a common incidental finding at imaging exams such as computed tomography (CT) and magnetic resonance imaging performed for unrelated reasons. Encompassing both benign and malignant entities, these lesions can prove challenging to classify. 2-[^18^F]FDG PET-CT is a recognized imaging modality for the characterization of indeterminate adrenal masses, but it is an expensive imaging modality and involves radiation exposure. To reduce the number of required scans and identify a less invasive potential alternative, we investigated whether CT radiomics could be used to predict the diagnostic parameter obtained using 2-[^18^F]FDG PET-CT (namely SUVmax). However, in our retrospective cohort of 179 adrenal masses and 150 PET-CT scans (of which 66 without iodine contrast injection), no correlation was found between the radiomics synthetic value (RadSV) and 2-[^18^F]FDG PET-CT SUVmax. This preliminary finding suggests that it might not be possible to use CT radiomics to reduce 2-[^18^F]FDG PET-CT referrals, confirming the role as problem solving tool of this imaging modality.

**Abstract:**

Background: Indeterminate adrenal masses (AM) pose a diagnostic challenge, and 2-[^18^F]FDG PET-CT serves as a problem-solving tool. Aim of this study was to investigate whether CT radiomics features could be used to predict the 2-[^18^F]FDG SUVmax of AM. Methods: Patients with AM on 2-[^18^F]FDG PET-CT scan were grouped based on iodine contrast injection as CT contrast-enhanced (CE) or CT unenhanced (NCE). Two-dimensional segmentations of AM were manually obtained by multiple operators on CT images. Image resampling and discretization (bin number = 16) were performed. 919 features were calculated using PyRadiomics. After scaling, unstable, redundant, and low variance features were discarded. Using linear regression and the Uniform Manifold Approximation and Projection technique, a CT radiomics synthetic value (RadSV) was obtained. The correlation between CT RadSV and 2-[^18^F]FDG SUVmax was assessed with Pearson test. Results: A total of 725 patients underwent PET-CT from April 2020 to April 2021. In 150 (21%) patients, a total of 179 AM (29 bilateral) were detected. Group CE consisted of 84 patients with 108 AM (size = 18.1 ± 4.9 mm) and Group NCE of 66 patients with 71 AM (size = 18.5 ± 3.8 mm). In both groups, 39 features were selected. No statisticallyf significant correlation between CT RadSV and 2-[^18^F]FDG SUVmax was found (Group CE, *r* = 0.18 and *p* = 0.058; Group NCE, *r* = 0.13 and *p* = 0.27). Conclusions: It might not be feasible to predict 2-[^18^F]FDG SUVmax of AM using CT RadSV. Its role as a problem-solving tool for indeterminate AM remains fundamental.

## 1. Introduction

Due to the widespread use of cross-sectional imaging in daily clinical practice, adrenal masses (AM) have become a common finding, being incidentally discovered in around 5% of patients undergoing a computed tomography (CT) scan for unrelated reasons [1,2]. Most of these lesions will prove to be benign (e.g., adrenal adenomas) and will not require further imaging nor treatment; however, the percentage of malignant AM (e.g., primary tumors, metastasis) is not negligible, and the risk increases in patients with known oncologic history [3]. While there are recognized magnetic resonance imaging (MRI) and CT imaging features (e.g., low attenuation on unenhanced CT or signal drop on out-of-phase chemical shift imaging) that can guide the diagnosis, this often proves complex due to atypical presentation and benign lesion mimics as well as technical and interpretative imaging pitfalls [4,5,6]. Uncertainty and variability in management decisions can lead to repeated exams for AM, which currently represent a significant economic burden on healthcare systems [7,8]. In the setting of indeterminate AM or when there is suspicion for metastatic disease, imaging guidelines recommend the use of 2-[^18^F]fluoro-2-deoxy-D-glucose (2-[^18^F]FDG) PET-CT as a reliable problem-solving tool that contributes in reducing the need for imaging-guided adrenal biopsies [9]. Indeed, 2-[^18^F]FDG PET-CT has shown a remarkable diagnostic accuracy for the characterization of AM, with both sensitivity and specificity values reported to be over 90% in two meta-analyses [10,11]. Regarding the assessment of metabolic activity for AM characterization, different maximum standardized uptake value (SUVmax) cut offs have been proposed [12,13].

Recently, there has been a growing interest around radiomics applications for the characterization of AM [14]. Radiomics is a promising post-processing technique that allows to obtain quantitative descriptors from medical images, among which novel imaging biomarkers of clinical relevance might be found [15]. Recent studies suggest that both MRI and CT radiomics might bring an added value in the discrimination between benign and malignant AM [16,17]. However, the methodological quality of radiomics studies on AM is heterogeneous, and the generalizability of radiomics-based decision support tools remains uncertain [18]. In this study, with an indirect approach to diagnosis and the objective to reduce the number of 2-[^18^F]FDG PET-CT referrals for AM evaluation, we aimed to assess the feasibility of CT radiomics to provide a synthetic parameter correlating with the SUVmax.

## 2. Materials and Methods

### 2.1. Study Population

Consecutive patients with AM who underwent a of 2-[^18^F]FDG PET-CT at our institution between April 2020 to April 2021were retrospectively enrolled. The study was performed in accordance with the ethical standards of the institutional research committee and with the principles of the 1964 Declaration of Helsinki and its later amendments (approved by the Institutional Review Board of University of Naples Federico II, Italy PG/2021/0034768, 7 April 2021).

The images were retrieved from the local picture archiving and communication system, while the SUVmax values of AM were obtained from the nuclear physician original reports. Exclusion criteria were reports or images not available, presence of artefacts (e.g., movement) at the level of adrenal glands in CT images, and AM with a maximum diameter < 1 cm. Based on whether iodine contrast agent had been administered, CT scans were divided in two groups, namely, enhanced (CE) and non-enhanced (NCE).

To determine the nature of included AM for descriptive purposes, the following criteria were applied according to availability: (1) Histopathological results; (2) Clinical-imaging follow-up (≥12 months); (3) Radionuclide studies; (4) Unenhanced CT mean Hounsfield Unit <10 (diagnostic criteria of lipid-rich adenoma) [19]; (5) CT morphologic features of malignancy (i.e., maximum diameter > 4 cm, inhomogeneous attenuation, and irregular margins, as assessed by an experienced radiologist).

### 2.2. Imaging Technique

2-[^18^F]FDG PET-CT were acquired using a Gemini TF 64 scanner (Philips Healthcare, Best, The Netherlands) as previously described [20]. At least 6 h of fasting were required from patients before image acquisition. Glycemia values were recorded to ensure <180 mg/dL at the time of radiotracer injection. 3D mode PET scan acquisitions started 60 min after 2-[^18^F]FDG injection (activity range tailored to body weight, ranging from 200 to 300 MBq). Low (70 mA) and high (230 mA) dose CT images were acquired (rotation time 1.5 s, collimation 16 × 0.625) for attenuation correction of emission data. SUVmax was measured according to state-of-the-art clinical practice by different experienced nuclear physicians.

### 2.3. Image Segmentation

**Figure 1:**
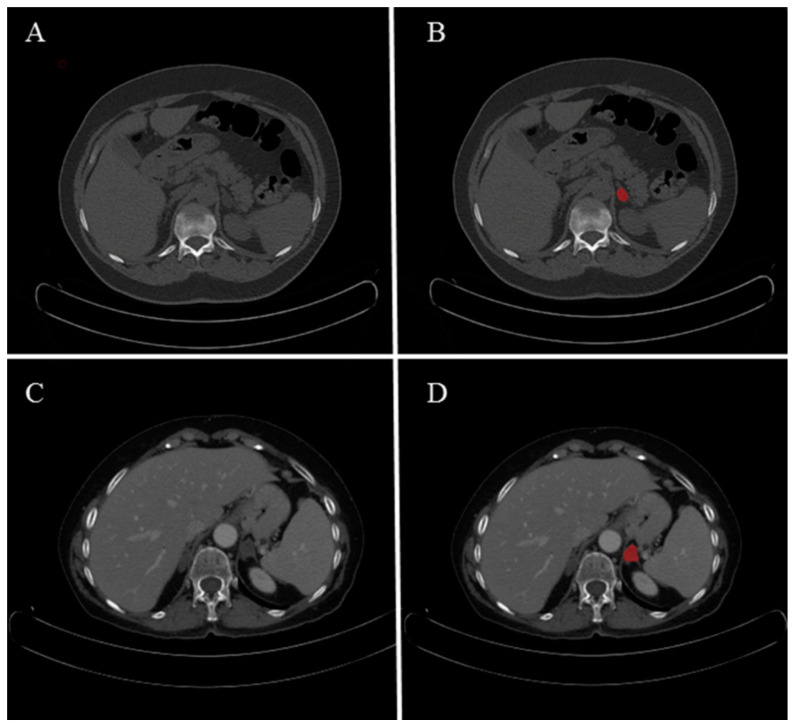
Segmentation example for unenhanced (**A**,**B**) and contrast-enhanced (**C**,**D**) CT. Images on axial plane with ROIs in red.

CT images obtained from the included 2-[^18^F]FDG PET-CT studies were reviewed on a dedicated software (RadiAnt DICOM Viewerv2021.2, https://www.radiantviewer.com accessed on 17 January 2022) to identify the AM described in the original reports. Then, using a segmentation tool (ITK-SNAPv3.6.0, http://www.itksnap.org/pmwiki/pmwiki.php, accessed on 17 January 2022) a final-year radiology resident manually annotated the CT images to obtain 2D regions of interest (ROI) for each AM in the two groups. The ROI was drawn at the slice in which the AM was larger and more conspicuous, taking care to encompass the entire AM as demonstrated on the slice. Then, to allow feature stability testing, a random sample of 30 AM was selected in each group, and two different operators with similar experience were asked to independently produce two additional sets of ROIs, one each. An example of lesion segmentation is showed in Figure 1.

### 2.4. Image Preprocessing and Radiomics Feature Extraction

For all the included CT scans, images were firstly resampled to isotropic pixel (1 × 1 mm). A fixed bin count of 16 was selected for image discretization. Feature extraction was performed using the PyRadiomics library (https://pyradiomics.readthedocs.io/en/latest/, v3.0), a tool for radiomics analysis that is compliant with the Imaging Biomarker Standardization Initiative (IBSI) [21]. From each ROI, 919 features were extracted, including first- and higher-order parameters but excluding shape features due to the characteristics of the segmentation process. Features were calculated both on the original images as well as after applying filters (i.e., wavelet decomposition and Laplacian of Gaussian).

### 2.5. Data Preprocessing and Feature Selection

Scaling was applied to bring feature values in a 0 to 1 range. Dimensionality reduction and feature selection were based on a multi-step process. First, features unstable to repeated segmentation were discarded. Specifically, a lower bound 95% confidence interval Intraclass Correlation Coefficient (ICC) analysis was performed, and features with ICC < 0.75 were deemed unstable. Then, features exhibiting low variance (<0.01) were identified with variance analysis and excluded. Finally, using an intercorrelation matrix redundant, highly intercorrelated (>0.8) features were detected and excluded from all subsequent steps.

### 2.6. Linear Regression and Radiomics Standardized Value (RadSV) Estimation

Using the remaining features and linear regression, the RadSV was calculated. Uniform Manifold Approximation and Projection (UMAP) was used to for dimensionality reduction of the radiomics dataset. This technique transforms the data to lower dimensionality projections and has proven useful in medical data analysis [22,23,24]. Specifically, the feature set obtained from the previously described analyses was projected to a one-dimensional space to obtain the Radiomics Standardized Value (RadSV) score. The correlation between RadSV and SUVmax of AM was evaluated with Pearson’s correlation coefficient (*r*).

## 3. Results

From a total of 725 patients who underwent 2-[^18^F]FDG PET-CT at our institution in the selected time interval, the final study population included 150 patients (179 AM), with 66 scans in the NCE (71 AM) and 84 in the CE (108 AM) group, respectively. Patients and AM clinical details can be found in Table 1 and Table 2. Briefly, the vast majority of patients were referred to 2-[^18^F]FDG PET-CT due to an oncologic disorder (among the most frequent in both groups, there were lung cancer and non-Hodgkin lymphoma). Most AM were benign in both groups (82/108, 76% in CE and 60/71, 85% in NCE group, respectively), with the prevalent diagnosis being adrenal adenoma (81/108, 75% in CE and 58/71, 82% in NCE group, respectively). Furthermore, five and twenty-two patients had more than one lesion, respectively, in NCE and CE group. Left-sided AM were prevalent in both groups (79% in NCE and 70% in CE group).

Of the 919 extracted features (per AM analysis), 779 (85%) were found to be unstable to repeated segmentation at ICC analysis and were thus discarded. None of the remaining 140 features stable features exhibited low variance, while another 72% (101/140) were highly intercorrelated and thus excluded from the analysis. The results were the same in both groups (NCE and CE) with a final number of 39 radiomics features left for the linear regression. A complete list of the 39 included features can be found in Table S1.

**Figure 2:**
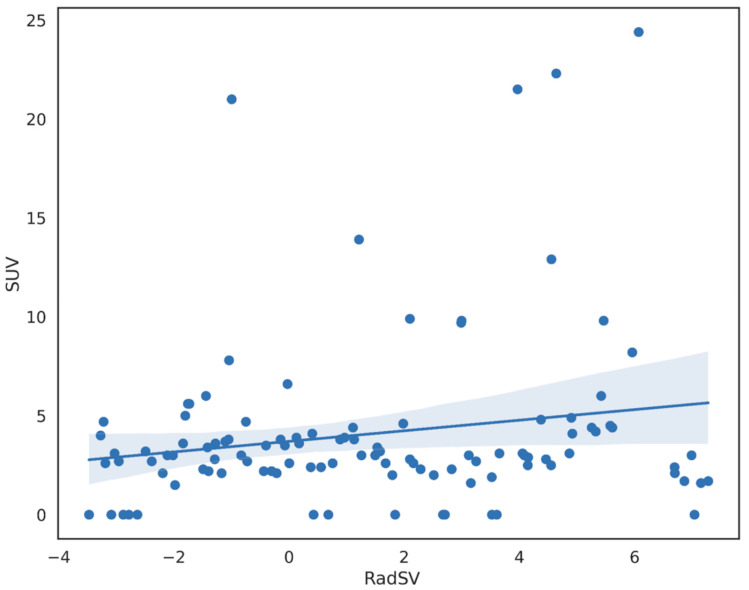
Plot for the linear regression model in the contrast-enhanced CT group.

**Figure 3:**
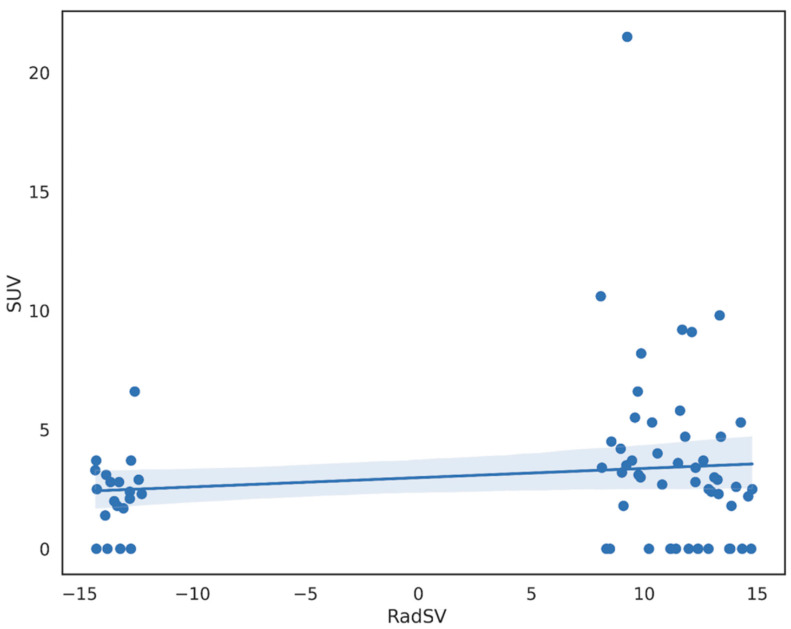
Plot for the linear regression model in the unenhanced CT group.

The obtained results are graphically shown in Figure 2 for CE group and Figure 3 for NCE group. In particular, no statistically significant correlation was found between the CT RadSV and the 2-[^18^F]FDG PET-CT SUVmax in both groups (NCE *r* = 0.13, *p* = 0.27; CE *r* = 0.18, *p* = 0.06).

## 4. Discussion

The diagnostic benefit of 2-[^18^F]FDG PET-CT for AM characterization derives from the in vivo assessment of lesion’s metabolism but entails additional costs for healthcare systems as well as further radiation exposure for patients. In line with the radiomics’ rational, which is that the heterogeneity at pixel level in medical images reflects the biological heterogeneity of cancer [15], we hypothesized that density and texture heterogeneity at CT could match with biological features correlating with the metabolic activity of AM. If successfully tested, such a hypothesis could have led to a non-invasive and cheaper alternative to 2-[^18^F]FDG PET-CT for AM characterization being available. Unfortunately, the RadSV obtained in this feasibility study from both contrast-enhanced and unenhanced CT images did not show a significant correlation with the metabolic activity (SUVmax) of AM. It could be speculated that, even at the pixel level, CT data simply do not contain information regarding AM metabolism. Regarding contrast-enhanced CT, a correlation between tumor vascularity and glucose consumption might have been expected, although it has been found that this is not the case for all cancers [25] and might not be contributing to the diagnostic value of RadSV in this setting. Furthermore, it should also be considered that an overlap between SUVmax values exhibited by benign and malignant AM has been reported [26]. This could lead to AM showing different radiomics signatures due to their different nature being classified as different in terms of metabolic activity despite showing a similar SUVmax value, de facto generating a confounding effect for the RadSV. Indeed, CT radiomics has shown a potential for the classification of indeterminate adrenal lesions in previous studies [27,28]. In particular, most studies were focused on the potential of radiomics features in discriminating between malignant and benign AM [29,30,31]. However, there are conflicting results on the accuracy of CT radiomics models for this classification task have been reported. Of note, a recent retrospective study on 160 patients affected by lung cancer with AM and biopsy as reference standard found that radiomics does not appear to have a possible clinically relevant role for AM characterization [31]. Using the same software (TexRAD) in a latter study, a previous investigation on 265 histologically confirmed adrenal masses built a support vector machine model using CT radiomics features that reached a diagnostic accuracy of 77% in the identification of adrenal metastases from benign AM (lipid-poor adenomas and pheochromocytomas) [29]. On the other hand, the number of studies focused on PET-CT radiomics applications for AM is quite limited at present, and the majority focuses on the analysis of PET images and the heterogeneity of metabolic activities patterns [32,33,34]. In particular, Nakajo et al. found that combining SUVmax with PET radiomics features such as homogeneity and entropy could increase the diagnostic accuracy of 2-[^18^F]FDG PET-CT in the identification of malignant AM [34]. Specifically, malignant AM showed higher SUVmax and entropy and lower homogeneity values compared to benign AM. However, while a more recent study from a different research group confirmed that PET textural parameters are significantly different between malignant and benign AM, they did not show any additional diagnostic value in combination with conventional PET parameters, thus partly conflicting with previous evidence [33].

In the field of radiomics, the presence of a strong publication bias that leads to neglecting negative results has been acknowledged [35,36]. On one hand, positive results encourage research efforts and can lead to scientific progress. However, radiomics is facing a reproducibility crisis, and the misconception that radiomics is always a feasible and useful approach should be challenged [37]. In this light, we believe that our work has the merit to present negative results reminding us that radiomics should bring added value to diagnostic imaging but is meant to be a complement, not a surrogate, to the already recognized approaches. On the other hand, it is recognized that SUVmax of 2-[^18^F]FDG PET-CT may overlap in benign and malignant AM due to different pathophysiological mechanisms causing increased FDG uptake [38]. Thus, testing the correlation between CT radiomics and SUVmax in subgroups of histologically different AM might be considered as the next step to validate and confirm our findings. Unfortunately, due to the marked prevalence of benign AM and particularly adrenal adenomas in our retrospective cohort (generating an unbalanced dataset) as well as the lack of a robust reference standard to minimize the risk of classification bias (i.e., histology), we could not perform such analysis in the present preliminary study. For the same issues, it was not possible to build a radiomics signature or train a machine learning algorithm to classify AM as either benign or malignant in our cohort. According to a recent systematic review, CT radiomics might indeed be a feasible tool to characterize AM [39]. However, going into details, eight out of the nine studies included in this systematic review did not perform radiomics feature stability testing to repeated segmentations, thus presenting a risk of having included unstable features in the proposed models. Furthermore, their approaches are highly heterogeneous from a methodological point of view (e.g., segmentation strategy, feature extraction, feature selection, and modeling), and without validation studies, the risk of overly optimistic estimates of accuracy cannot be neglected.

The present study is not free from limitations, and these deserve to be acknowledged. Firstly, alternative approaches (e.g., 3D image segmentation) were not explored, and it might be questioned whether including more pixels in the analysis could have led to better results for the RadSV. However, the superiority of a 3D approach for radiomics is not yet established both in terms of feature reproducibility and resulting model performance [40,41]. Also, the low correlation values obtained do not suggest a realistically marginal improvement in performance and would have led to significantly better findings. A cut-off-based analysis of SUVmax could have also been performed, but since a clear cut-off value has not been established, using SUVmax as a continuous variable appeared to be the most sensible choice. The retrospective design brings an inherent risk of selection bias, and a more diverse (possibly from multiple centers) and bigger sample might have been a better representative of the target population. SUV ratios (liver or blood pool) have been proposed as a better alternative to SUVmax for AM characterization, representing a possible subject of investigation [42]. The high prevalence of adrenal adenomas might also represent a possible source of bias and deserves further investigation. Finally, since we used co-registered CT images, it remains partly unclear whether radiomics based on diagnostic CT images (e.g., from adrenal-CT protocols) might lead to different results. Nevertheless, the advantage of having the same time point acquisition for both CT radiomics and SUVmax is undeniable for this kind of analysis.

## 5. Conclusions

Our results indicate that the diagnostic role of 2-[^18^F]FDG PET-CT in AM characterization cannot be surrogated by CT radiomics as the RadSV does not correlate with the SUVmax. A more careful and standardized diagnostic pathway might be a better strategy to avoid unnecessary scans and costs.

## Figures and Tables

**Table 1:** Main characteristics of the study population (patients).

	NCE Patients (*n* = 66)	CE Patients (*n* = 84)
Age (years) *	68 (15)	67 (5)
Oncologic patients (%)	92	95
Primary tumor site (*n*)	13 lung cancer	14 non-Hodgkin lymphoma
	10 multiple myeloma	13 melanoma
	8 non-Hodgkin lymphoma	12 breast cancer
	3 breast cancer	9 lung cancer
	3 pancreatic cancer	6 pancreatic cancer
	3 colon cancer	4 colon cancer
	26 others (2 cases or less)	26 others (2 cases or less)

NCE: non contrast-enhanced, CE: contrast-enhanced. * Expressed as median with interquartile range in parenthesis.

**Table 2:** Main characteristics of the included adrenal masses.

	NCE Lesions (*n* = 71)	CE Lesions (*n* = 108)
Adenoma (*n*)	58	81
Pheochromocytoma (*n*)	2	1
Metastasis (*n*)	11	26
Multiple lesions (*n*)	5	24
Left side lesions (%)	79	70
Lesion maximum diameter (mm) ^#^	18.5 ± 3.8	18.1 ± 4.9
SUVmax ^#^	2.0 ± 1.7	3.8 ± 3.5

NCE: non contrast-enhanced, CE: contrast-enhanced. Histologic confirmation was available for five lesions. ^#^ Expressed as mean ± standard deviation.

## Data Availability

The datasets generated during and/or analyzed during the current study are available from the corresponding author on reasonable request.

## Supplementary Materials

The following supporting information can be downloaded at: https://www.mdpi.com/article/10.3390/cancers15133439/s1, Table S1: complete list of selected radiomics features. Reference [43] is cited in Supplementary Materials.
