# Supplementary material for: Prediction of 2-[18F]FDG PET-CT SUVmax for Adrenal Mass Characterization: A CT Radiomics Feasibility Study
Source: Cancers (Basel). 2023 Jun 30;15(13):3439. doi: 10.3390/cancers15133439 (PMC10340369; doi:10.3390/cancers15133439)
Supplement: Supplementary file 1 [file cancers-15-03439-s001.zip › cancers-2377688-supplementary.pdf]

Table S1. Complete list of selected radiomics features

original_firstorder_10Percentile
original_firstorder_InterquartileRange
original_firstorder_Range
original_firstorder_Skewness
original_firstorder_Uniformity
original_glcm_JointEntropy
original_glszm_LargeAreaEmphasis
log-sigma-1-0-mm-3D_firstorder_10Percentile
log-sigma-1-0-mm-3D_firstorder_90Percentile
log-sigma-1-0-mm-3D_firstorder_Entropy
log-sigma-1-0-mm-3D_firstorder_Skewness
log-sigma-1-0-mm-3D_firstorder_TotalEnergy
log-sigma-1-0-mm-3D_glrlm_RunLengthNonUniformityNormalized
log-sigma-1-0-mm-3D_gldm_LargeDependenceHighGrayLevelEmphasis
log-sigma-2-0-mm-3D_firstorder_Kurtosis
log-sigma-2-0-mm-3D_glrlm_LongRunHighGrayLevelEmphasis
log-sigma-3-0-mm-3D_firstorder_InterquartileRange
log-sigma-3-0-mm-3D_firstorder_Mean
log-sigma-4-0-mm-3D_firstorder_Maximum
log-sigma-4-0-mm-3D_firstorder_Minimum
log-sigma-5-0-mm-3D_firstorder_MeanAbsoluteDeviation
wavelet-LH_firstorder_10Percentile
wavelet-LH_firstorder_90Percentile
wavelet-LH_firstorder_Kurtosis
wavelet-LH_firstorder_Maximum
wavelet-LH_firstorder_Minimum
wavelet-LH_glcm_JointEnergy
wavelet-LH_glszm_LargeAreaEmphasis
wavelet-LH_glszm_LargeAreaLowGrayLevelEmphasis
wavelet-HL_firstorder_10Percentile

wavelet-HL_firstorder_Kurtosis
wavelet-HL_firstorder_Minimum
wavelet-HL_gldm_LargeDependenceHighGrayLevelEmphasis
wavelet-HH_firstorder_10Percentile
wavelet-HH_firstorder_Kurtosis
wavelet-HH_firstorder_Minimum
wavelet-HH_glcm_MaximumProbability
wavelet-HH_glrlm_LongRunEmphasis
wavelet-HH_glszm_LargeAreaLowGrayLevelEmphasis

Definition and formulas for the radiomics features can be found in the PyRadiomics online documentation (https://pyradiomics.readthedocs.io/en/latest/features.html).

Interestingly, most of the selected features were extracted from filtered images rather than original ones, which is in line with a recent study suggesting that filters should be adopted in radiomics analysis [43].

On the other hand, the majority of selected features are from the first order category. These are statistics features that describe the distribution of voxel intensities within the image region defined by the mask through commonly used and basic metrics. In case of positive findings, this might have aided in understanding the mechanics behind the predictions of the model, as such features are more easily defined.
